# Characterization of SARS-CoV-2-specific antibodies in COVID-19 patients reveals highly potent neutralizing IgA

**DOI:** 10.1038/s41392-021-00478-7

**Published:** 2021-01-29

**Authors:** Weihong Zeng, Huan Ma, Chengchao Ding, Yunru Yang, Yong Sun, Xiaoxue Huang, Weihuang He, Yan Xiang, Yong Gao, Tengchuan Jin

**Affiliations:** 1 Department of Obstetrics and Gynecology, The First Affiliated Hospital of USTC, Division of Life Sciences and Medicine, University of Science and Technology of China, Hefei, Anhui China; 2 Hefei National Laboratory for Physical Sciences at Microscale, CAS Key Laboratory of Innate Immunity and Chronic Disease, Division of Life Sciences and Medicine, School of Basic Medical Sciences, University of Science and Technology of China, Hefei, Anhui China; 3 Division of Life Sciences and Medicine, Department of Pulmonary and Critical Care Medicine, The First Affiliated Hospital of USTC, University of Science and Technology of China, Hefei, Anhui China; 4 Division of Life Sciences and Medicine, Department of Infectious Diseases, The First Affiliated Hospital of USTC, University of Science and Technology of China, Hefei, Anhui China; 5 Anhui Center for Disease Control and Prevention (Anhui CDC), Hefei, Anhui China; 6 Kangrun Biotech LTD, Guangzhou, Guangdong China; 7 Department of Microbiology, Immunology and Molecular Genetics, University of Texas Health Science Center at San Antonio, San Antonio, TX USA; 8 CAS Center for Excellence in Molecular Cell Science, Chinese Academy of Science, Shanghai, China

**Keywords:** Infectious diseases, Structural biology

**Dear Editor**,

The coronavirus disease 2019 (COVID-19), caused by the Severe Acute Respiratory Syndrome Coronavirus 2 (SARS-CoV-2), is an ongoing threat to public health. Due to the presence of neutralizing antibodies in convalescent patients, convalescent plasma transfusion has been tested as a promising therapy for severe COVID-19 patients.^1,2^ We and others previously found a surprisingly early serum IgA antibody response in the COVID-19 patients.^3,4^ However, the relative contribution of the IgA antibody towards virus neutralization with respect to other antibody isotypes is unknown.

Highly purified SARS-CoV-2 spike receptor-binding domain (RBD) and Nucleocapsid protein (NP) (Supplementary Fig. S1) were employed to develop affinity immobilized columns, respectively. Serum antibodies specific to RBD and NP were purified from 90 mL virus-inactivated sera collected from 50 COVID-19 convalescent patients using the columns prepared above, respectively (Supplementary Fig. S2a, e). NP or RBD-specific IgG was then purified by using a protein G column, which only binds to IgG (Supplementary Fig. S2b, f). The NP or RBD-specific IgM were purified by using an anti-IgM affinity column and IgA was further purified by pull-down using anti-IgA affinity beads (Supplementary Fig. S2c, g), respectively (see Supplemental Methods for details). The purified NP or RBD-IgA, -IgM, and -IgG were confirmed by western blotting with isotype-specific antibodies (Supplementary Fig. S3) and mass spectrometry (data not shown). The amount of RBD- or NP-specific antibodies is in the order of IgG > IgA > IgM (Supplementary Table S1).

Absolute concentrations of each antibody isotypes provide important humoral immune response information in COVID-19 patients. Using these highly purified antibodies as standards, we generated standard curves (Supplementary Fig. S4) to convert the relative light unit (RLU) measured with RBD and NP based chemiluminescence diagnostic kits into absolute antibody concentrations (μg per mL) for 216 serum samples from 87 COVID-19 patients. To simplify plots from a large number of samples, median values with an interquartile range of antibody concentrations were plotted vs. time windows. As shown in Fig. 1a–c, the median concentration of RBD-IgA reached the highest (8.84 μg/mL) from 16 to 20 days after illness onset, declining subsequently but maintaining at about 3.62 μg/mL until 41 days. The median concentration of RBD-IgG was the lowest in the early stages but rise after the 15th day after the illness onset. RBD-IgG concentration reached the peak on the 21st–25th days after illness onset at 16.47 μg/mL and stayed at a relatively high concentration (11.40 μg/mL) until the 41st day. The trend of RBD-IgM was very similar to that of RBD-IgA. As expected, the trend of NP-specific antibodies was roughly the same as that of RBD-specific antibodies (Fig. 1d–f) for each isotype. The median concentration of NP-IgA reached the highest (10.07 μg/mL) level on the 11th–15th days after illness onset. The median concentration of NP-IgG reached the peak on the 21st–25th days after the illness onset as 149.37 μg/mL. Unlike NP-IgA, the highest median concentration of NP-IgM (6.72 μg/mL) appeared on the 16th–20th days after the illness onset. The absolute concentration of the three isotypes of antibody in 216 COVID-19 patients’ sera provides a guide for convalescent serum therapy and vaccine development.

**Fig. 1:**
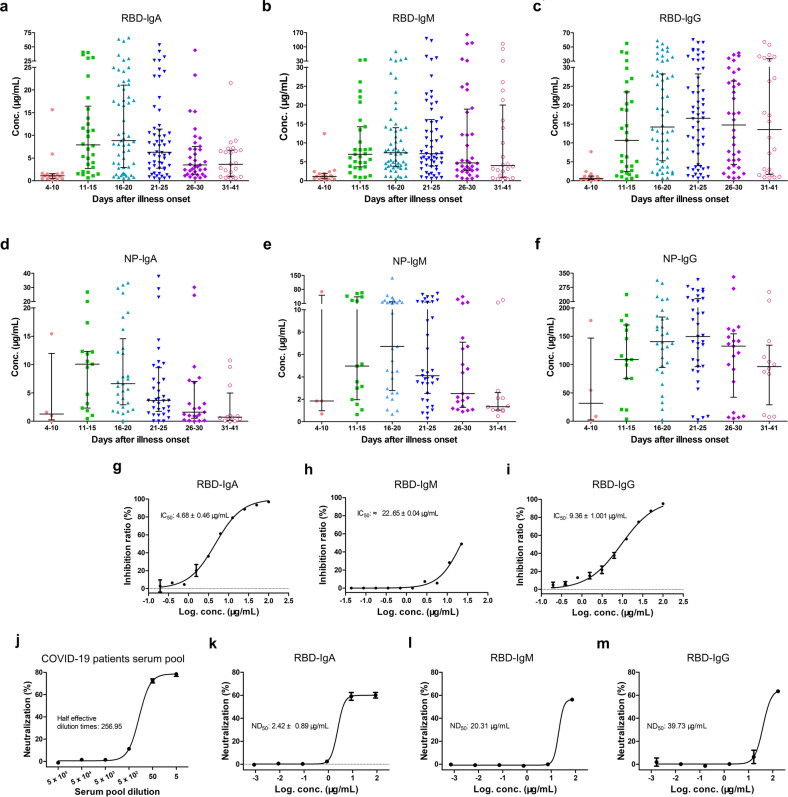
Characterization of serum IgA, IgM, and IgG in COVID-19 patients. **a**–**c** The kinetics of RBD-specific IgA (**a**), IgM (**b**), and IgG (**c**) levels in sera of COVID-19 patients at different time windows. **d**–**f** The kinetics of NP-specific IgA (**d**), IgM (**e**), and IgG (**f**) levels in sera of COVID-19 patients at different time windows. The median values of RLU or calculated antibody mass concentrations were plotted for each isotype of three antibodies. Bars indicate interquartile ranges. **g**, **h** The efficiency of RBD-IgA (**g**), IgM (**h**), and IgG (**i**) competing with hACE2 in binding SARS-CoV-2 RBD were determined by ELISA. The IC_50_ was measured by GraphPad Prism 5.0. **j**–**m** COVID-19 convalescent patients’ serum pool (**j**) and RBD-specific IgA (**k**)/IgM (**l**)/IgG (**m**)-mediated neutralization of SARS-CoV-2 infection in Vero-E6 cells. The ND_50_ were fitted by GraphPad Prism 5.0

Biolayer interferometry (BLI) was performed to further characterize the binding properties of the purified antibodies. As shown in Supplementary Fig. S5d–f, the purified SARS-CoV-2 NP-IgA, -IgM, and -IgG exhibited a high cross-reactivity with the SARS-CoV-1 NP, probably due to the high identity between SARS-CoV-2 NP and SARS-CoV-1 NP. By contrast, RBD-IgA, -IgM, and -IgG specifically bound to SARS-CoV-2 RBD but not SARS-CoV-1 RBD (Supplementary Fig. S5a–c). These results indicate that RBD is more suitable than NP as an antigen for specific testing kits.

SARS-CoV-2 virus entry into host cells requires the interaction between RBD and Angiotensin-converting enzyme 2 (ACE2) on the cell surface, so the RBD antibodies may block virus infection. Competitive ELISA was performed to assess the RBD-ACE2 blocking abilities of the RBD-specific antibodies. As shown in Fig. 1g–i, the three isotypes of RBD-specific antibodies block the binding of ACE2 to the RBD immobilized on plate with different strengths. Among them, IgA exhibited the strongest competitive capacity with IC_50_ of 4.68 ± 0.46 μg/mL, which was better than that of IgG (IC_50_ = 9.36 ± 1.001 μg/mL) and IgM (IC_50_ = 22.65 ± 0.04 μg/mL). Furthermore, the SARS-CoV-2 neutralizing assay was performed to evaluate the neutralizing potency of the RBD-specific antibodies. The sera pool of COVID-19 convalescent patients was tested first, which exhibited an 80% neutralizing ability of SARS-CoV-2 virus infection of Vero-E6 cells (Fig. 1j). Subsequently, the purified RBD-specific antibodies were tested separately. RBD-IgA, -IgM, and -IgG showed different SARS-CoV-2 infection neutralizing potency. Consistent with the results of competitive ELISA, IgA exhibited a higher neutralizing potency than IgM and IgG. The ND_50_ value of IgA was 2.42 ± 0.89 μg/mL, while ND_50_ values of IgM and IgG were nearly 20.31 μg/mL and 39.73 μg/mL, respectively (Fig. 1k–m). Even though IgA is only 2-fold stronger than IgG in competing ACE2 to bind with RBD, IgA is about 10-fold more potent than IgG in the neutralization assay. These results showed that the IgA antibodies present in convalescent patients’ sera play non-neglectable roles in neutralizing SARS-CoV-2 in addition to the traditionally thought IgG.

Studies on humoral responses to infections mediated by IgA are receiving increasing attention. It was proposed that secretory IgA might be a therapeutic strategy against SARS-CoV-2.^5^ However, there is no experimental data on anti-SARS-CoV-2 efficacy by IgA in serum. Here, we purified NP- and RBD-specific antibodies directly from convalescent patients’ serum pool, and determined the absolute mass concentration of all types of serum antibodies in COVID-19 patients. RBD-specific antibodies are able to block ACE2 binding to RBD and neutralize live SARS-CoV-2. RBD-IgA exhibited an impressive neutralizing potency, with an ND_50_ value of 2.42 ± 0.89 μg/mL, which is 10-fold better than that of RBD-IgG. This study provides direct evidence for the potent antiviral activity of serum-derived RBD-IgA. Furthermore, our results show that the serum pool from COVID-19 convalescent is more potent than individual purified RBD antibodies in terms of antiviral activities, suggesting the presence of other antibody blocking sites on the virus and additive effect of different antibodies, which offering a theoretic basis for convalescent serum therapy and combination antibody treatment strategy.

## Supplementary information

Characterization of SARS-CoV-2 Specific Antibodies in COVID-19 Patients Reveals Highly Potent Neutralizing IgA
