# Supplementary material for: Characterization of SARS-CoV-2-specific antibodies in COVID-19 patients reveals highly potent neutralizing IgA
Source: Signal Transduct Target Ther. 2021 Jan 29;6:35. doi: 10.1038/s41392-021-00478-7 (PMC7844101; doi:10.1038/s41392-021-00478-7)
Supplement: Supplementary file 1 — Characterization of SARS-CoV-2 Specific Antibodies in COVID-19 Patients Reveals Highly Potent Neutralizing IgA [file 41392_2021_478_MOESM1_ESM.pdf]

Supplementary Materials for

Characterization of SARS-CoV-2 Specific Antibodies in COVID-19 Patients Reveals Highly Potent Neutralizing IgA

This file includes:

Materials and Methods

Figures. S1 to S5

Tables S1

Materials and Methods

Serum Sample preparation

The details of 216 serum samples for antibodies detection from 87 COVID-19 patients were described before.[3] For serum antibodies purification, this study enrolled a total of 90 mL from 50 cases of COVID-19 patients admitted to the First Affiliated Hospital of USTC Hospital between Jan 30 and Feb 13, 2020, and their blood samples were collected during routine clinical testing in hospitalization and after discharged from the hospital. All enrolled cases were confirmed to be infected with SARS-CoV-2 using real time RT-PCR (RT-qPCR) test on throat swab samples from the upper respiratory tract. Final concentration of a denaturant solution containing 1% TNBP and 1% Triton X-100 was added to the serum pool. After adequate mixing by inverting, the sample was incubated at 30 ℃ for 4 hours to completely inactivate any potential viruses. Such solvent/detergent treatment is recommended by WHO guidelines on viral inactivation and removal procedures intended to assure the viral safety of human blood plasma products (https://www.who.int/bloodproducts/publications/WHO_TRS_924_A4.pdf).

Molecular cloning, protein expression and purification

The expression and purification of nucleocapsid protein [(A1-A419) (NCBI accession code: ADI66791.1)] in *E. coli* was performed as described previously (doi: 10.1016/j.bbrc.2020.04.136.). Briefly, special treatment during the addition of high salt in lysis buffer and a hydrophobic interaction column was used to completely remove non-specific nucleic acid contamination. The final protein was homogeneous and free of nucleic acid contamination as revealed by gel filtration and UV-Vis spectrum.

To make recombinant SARS-CoV-2 RBD [(A321-A591) (NCBI accession code: QLI52045)], SARS-CoV RBD [(A309-A540) (NCBI accession code: YP_009825051.1)] and hACE2 [(A19-A615) (NCBI accession code: NP_001358344.1)], an IFNA1 leader sequence, a sequence encoding receptor binding domain of spike protein or extracellular domain of ACE2 and a human IgG1-Fc was fused together in this order and cloned into pTT5 vector. The construct was transiently transfected into HEK293F cells by polyethylenimine (Polyscience). After three days of expression, fusion protein was purified from cell supernatant using protein A column (GE Healthcare).

Preparation of Antigen immobilized affinity columns

The N protein or RBD protein was coupled to agarose resin (CNBr-activated Sepahrose 4B) according

to the manufacturer's protocols (GE Healthcare). Briefly, 1.75 g of lyophilized powder was suspended in 100 mL of 1 mM HCl and washed using a MACS Mix for 15 min, and the supernatant was removed after centrifugation. 10 mg of protein was first diluted in 5 mL of coupling buffer (0.1 M $NaHCO_3$ pH 8.3, 0.5 M NaCl), then added to the well-washed resin. The mixture was incubated by MACS Mix overnight at 4 ℃. The protein-coupled resin was packed into a 5 mL empty column, washed 5 times withcoupling buffer to remove the excess protein and then blocked the active group by loading 0.1 M Tris-HCl. The packaged columns were ready to be used after alternately washing by acid buffer (0.1 M acetic acid/sodium acetate pH 4.0, 0.5 M NaCl) and alkaline buffer (0.1 M Tris pH 8.0, 0.5 M NaCl), respectively.

Purification of IgG，IgM and IgA of N and RBD protein

Ammonium sulfate powder was added to the serum to a final concentration of 3 M. After stirring at room temperature for 15 min or till completely dissolved, the suspension was centrifuged at 13,000 rpm/min for 30 min at 4 ℃ to remove the lipids and supernatant. The pellets were re-suspended in 40 mL of PBS and filtered before loading onto an N protein affinity column. The sample was eluted with a linear gradient of elution buffer (0.1 M HAcO) and further purified by a protein G column with elution buffer (0.1 M HAcO). Both of the flow-through and elution peak (IgG) from protein G column were collected. Then the flow-through sample was further purified by an anti-IgM column and the bound protein was eluted. The flow-through from anti-IgM column was corresponding to IgA while the eluted peak was corresponding to IgM.

The flow-through fraction from the N protein affinity column was loaded onto an RBD-Fc affinity column and eluted with a linear gradient of elution buffer (0.1 M HAcO). The IgG, IgM and IgA of the spike protein RBD were then purified using a protein G column and an anti-IgM column following the same procedure as that of purifying IgG, IgM and IgA of N protein. After purification, all isolated antibodies were verified by SDS-PAGE and identified by mass spectrum.

Western Blot

The purified antibodies of IgG, IgM, IgA against NP and RBD were boiled in the reduced SDS loading buffer for 3 min. The supernatant was loaded onto an SDS-PAGE gel along with a molecular weight marker. The protein was transferred from the PAGE gel to a PVDF membrane (Millipore). The membrane was incubated in blocking buffer [5% defatted milk (w/v) in TBST (0.1% Tween 20, 150 mM NaCl, 20 mM Tris-HCl pH 7.5)] for 1 h at room temperature. Subsequently, the membrane was incubated with HRP labeled anti-IgG-Fc (1:5000, Sino Biological)/anti-IgA (1:4000, Boster Biological)/anti-IgM-μ (1:6000, Boster Biological) secondary antibody in blocking buffer for 1 hr at room temperature. Lastly, the membrane was washed by TBST again, and detected by ECL kit (abpbiotech) using chemiluminescence apparatus (Bio-Rad).

NP and RBD-specific isotype antibodies standard detection using chemiluminescence assay

Purified NP and RBD-specific subtype antibodies were serially diluted using dilution buffer (Kangrun Bio.tech). Then the diluted antibodies were detected by chemiluminescence instrument (Kangrun Bio.tech). The data was analyzed by Microsoft Excel software.

Serological test of COVID-19 convalescent patients of NP and RBD-specific subtype antibodies using chemiluminescence assay

The collected serum samples of COVID-19 patients and were treated as described above. The serum was diluted 40 times by dilution buffer (Kang Run Biotech) then detected by chemiluminescence instrument (Kangrun Biotech). The data was analyzed by Prism 5.0.

Antigen-antibody interaction analysis by BLI

Molecular interactions were studied with a ForteBio RED96 system. The N and RBD proteins from either SARS-CoV-2 or SARS were immobilized to an AR2G sensor chip (FortéBio) using an RED96 system (FortéBio). AR2G sensor chip was wetted by DPBS for 10 min and then activated for 1500 s by the mixture of 0.4 M of EDC and 0.1 M NHS (1:1). The N and RBD proteins were diluted into 200 μL of NaAcO buffer (20 mM, pH 3.6 or pH 5.0) to a final concentration of 30 μg/mL respectively, then loaded onto the sensor for a duration of 2000 s to couple onto the sensor. Due to the low coupling efficiency, N protein was coupled twice. The sensor was washed with aminoethanol buffer (pH 8.5) to block excessive activated sites. Antibodies were diluted with PBS to 9 μg/mL, then flown on to the sensor. The association and dissociation curves were acquired. Results were analyzed by ForteBio Data Analysis software and Prism 5.0.

Competitive ELISA

The hACE2-Fc fusion protein was biotinylated by Sulfo-NHS-LC-LC-Biotin (Thermo) overnight at 4 °C. 2 μg/mL purified SARS-CoV-2 RBD proteins free of Fc tag was coated in a 96-well immune plate (Thermo) overnight at 4 ℃. The plate was washed by PBS then blocked with PBST containing 5% defatted milk (w/v) for 2 hr at room temperature. 10 nM biotinylated hACE2-Fc was mixed with serially diluted RBD-IgA or RBD-IgM or RBD-IgG antibodies (100 μg/mL, 50 μg/mL, 25 μg/mL…0.19 μg/mL, 0), and was then added to the plate and incubated for 1 hr at room temperature. After being washed by PBST (PBS containing 0.1 % Tween-20), the plate was incubated with HRP-labled Streptavidin (1:5000, Beyotime) for 30 min. After being washed for 5 times with PBST, 100 μL per well TMB (Beyotime) was added and reacted under dark for 10 min. Lastly, 50 μL of H_2SO_4 (1 M) was added to each well to stop the reaction. OD_{450} was read by a Synergy H1 plate reader (Biotek). The data was analyzed by Prism 5.0.

SARS-CoV-2 neutralization assay

Live SARS-CoV-2 was provided by the Centers for Disease Control and Prevention (CDC) of Anhui Province and the whole neutralization assays were performed in BSL-3 laboratory. Vero-E6 cells were seeded in 96-well plates for 1 day before infection when the cell density reached 85%. Ten fold serial dilutions were made for RBD-IgA, IgM and IgG from concentrations of 88 μg/mL, 72 μg/mL and 164.7 μg/mL, respectively. Each diluted antibody was mixed with an equal volume of SARS-CoV-2 at a 20 TCID50. The mixture was then added to Vero-E6 cells immediately and incubated for 1 hour in incubator. The cells were washed by PBS and further incubated in DMEM supplemented with 2% of FBS for 48 hours. The cells were then lysed and the SARS-CoV-2 nucleic acid was extracted by RNA extraction machine using RNA extraction kits (Tianlong Biotech).

Nucleic acid quantification was performed by RT-qPCR with the following primers targeting nucleocapsid gene: NP-for: 5’-GGG GAA CTT CTC CTG CTA GAA T-3’, NP-rev: 5’-CAG ACA TTT TGC TCT CAA GCT G-3’, probe: 5’ FAM-TTG CTG CTG CTT GAC AGA TT-TAMRA 3’ (Sangong Biotech). The results were analyzed using Prism 5.0.

Figure. S1.

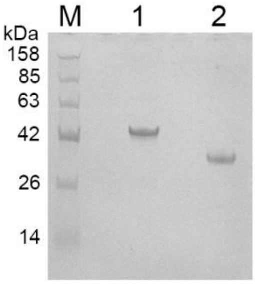

Fig. S1. Purification of recombinant SARS-CoV-2 Nucleocapsid protein and RBD. After SDS-PAGE separation based on molecular weight (kDa) together with protein size markers (M), the proteins were stained with Coomassie Blue. Predicted molecular mass of NP (1) and RBD (2) are 45.6 kDa and 30.3 kDa, respectively.

Figure. S2.

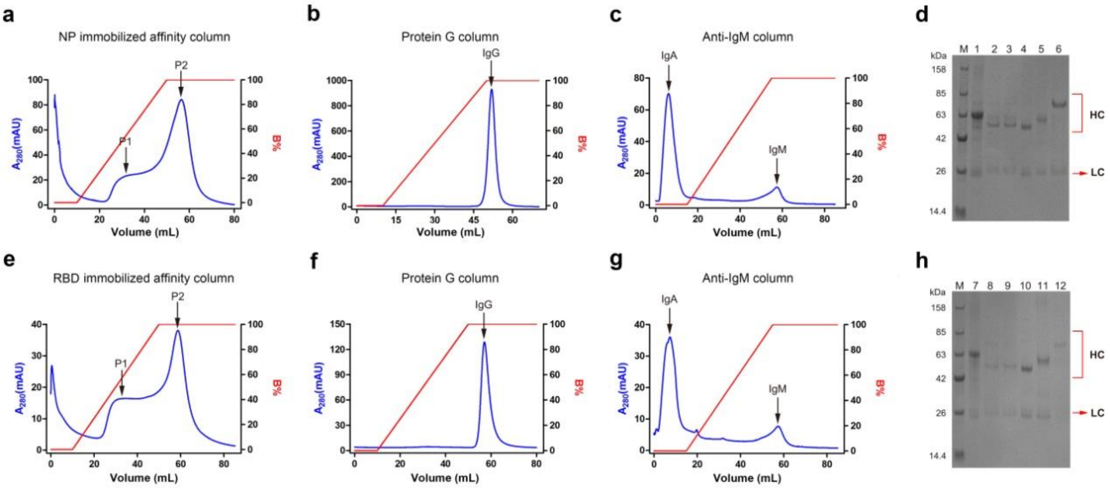

Fig. S2. Purification of anti-nucleocapsid and anti-RBD antibodies from convalescent COVID-19 patients' serum pool.

90 mL virus-inactivated COVID-19 convalescent patients' serum were loaded into NP-immobilized column, the NP-specific antibodies were linear eluted by acetic acid (**a**).The eluted antibodies were then applied to protein G column, the NP-IgG were linear eluted by acetic acid (**b**). The flowthrough of protein G column were purified by anti-IgM column, the NP-IgM were linear eluted by acetic acid (**c**) and the flowthrough were NP-IgA. NP-IgA were further purified by anti-IgA beads. The isolated NP-specific antibodies were verified by SDS-PAGE (**d**). The RBD-specific antibodies were purified by RBD-immobilized column from the 90 mL serum (**e**). The RBD-IgG (**f**) and RBD-IgM (**g**) were isolated by protein G column and anti-IgM column. RBD-IgA were also further purified by anti-IgA beads. The isolated RBD-specific antibodies were verified by SDS-PAGE (**h**).

M: marker; 1: total protein in serum; 2-3: P1 and P2 purified by NP-immobilized affinity column; 4:

IgG purified by Protein G column; 5-6: IgA and IgM purified by anti-IgM column; HC: heavy chain; LC: light chain.

Figure. S3.

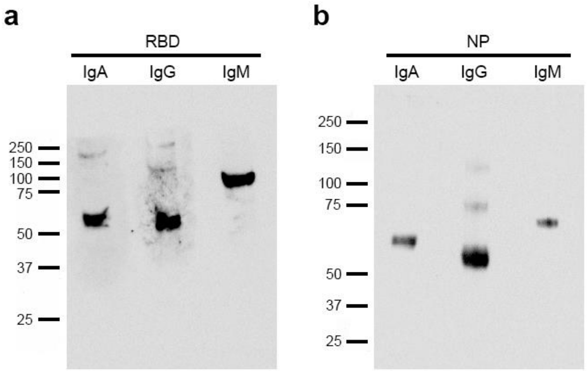

Fig. S3. Verification of purified antibodies.

Purified different isotypes of antibodies were verified by western blotting using HRP-anti-human IgA-Fc/ IgM-μ chain/ IgG-Fc secondary antibodies.

a The heavy chains of NP-specific antibodies were verified by HRP-conjugated secondary antibodies.

b The heavy chains of RBD-specific antibodies were verified by HRP-conjugated secondary antibodies.

Figure. S4.

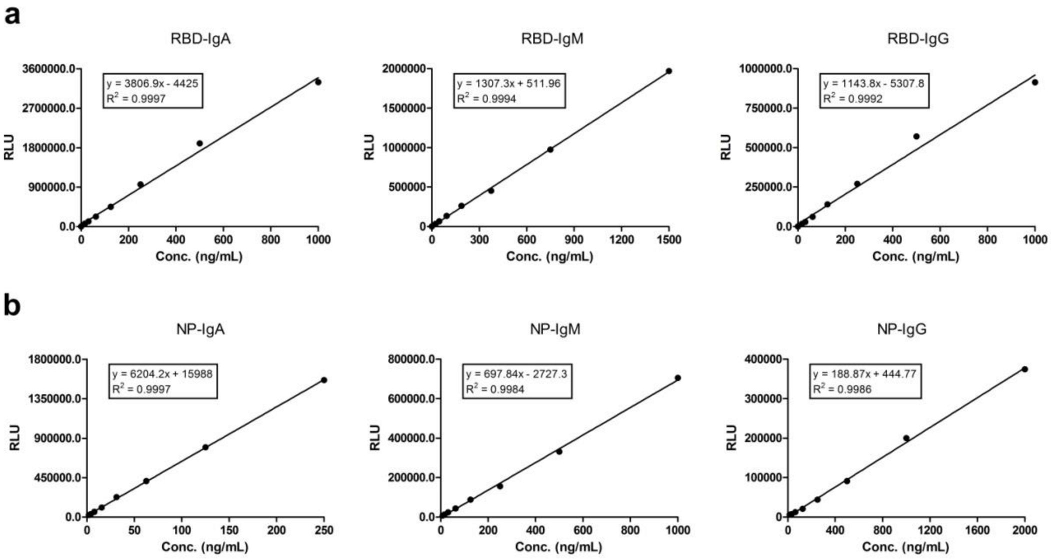

Fig. S4. Standard curves for RBD-and NP-specific antibodies.

a Standard curves were constructed based on immunoassay of 8 concentrations of RBD-specific IgA, IgM or IgG antibodies.

b Standard curves were constructed based on immunoassay of 8 concentrations of NP-specific IgA, IgM or IgG antibodies.

Figure. S5.

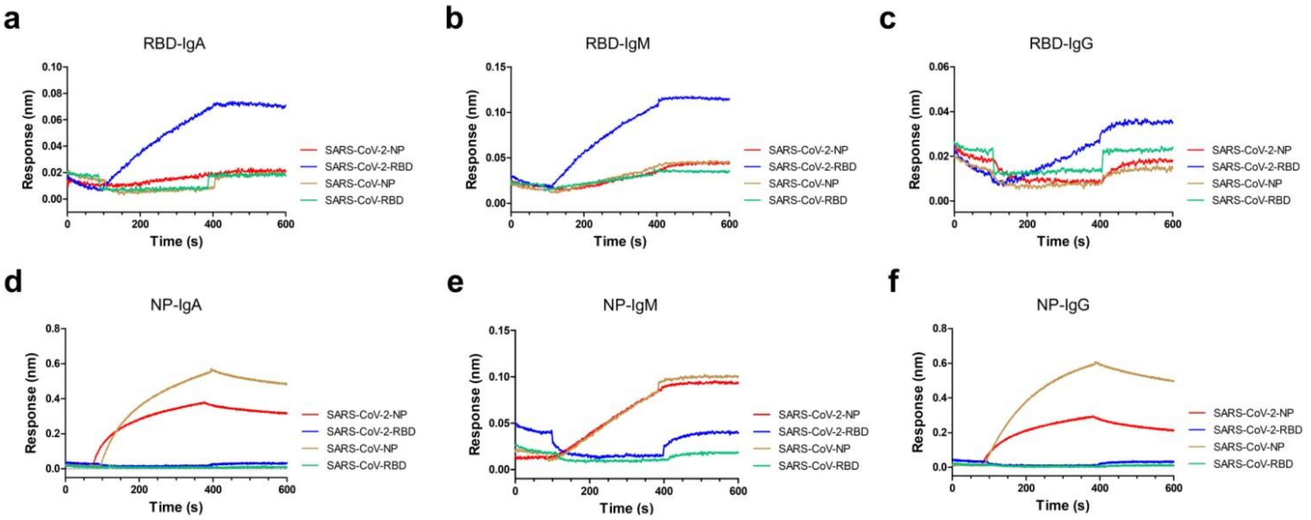

Fig. S5. Specificity evaluation of serum antibodies targeting different antigens.
Specific binding profiles of SARS-CoV-2 RBD/ SARS-CoV RBD/ SARS-CoV-2 NP/ SARS-CoV NP to RBD-IgA (**a**), RBD-IgM (**b**) and RBD-IgG (**c**), or NP-IgA (**d**), NP-IgM (**e**) and NP-IgG (**f**) by ForteBio. The curves fitted by GraphPad Prism 5.0.

Table S1.

Quantity of SARS-CoV-2 antibodies and their affinities

**Antigen and**	**RBD**			**Nucleocapside Protein**		
**antibody isotype**	**IgA**	**IgM**	**IgG**	**IgA**	**IgM**	**IgG**
Quantities from 90 mL sera (mg)	0.736	0.11	3.62	0.895	0.115	9.52
